# The impact of chronic comorbidities on cancer immunoediting: challenges and opportunities for immunotherapies

**DOI:** 10.3389/fimmu.2026.1773872

**Published:** 2026-03-06

**Authors:** Kassandra Ofelia Rodríguez-Aguillón, Mónica Lizeth González-González, Kenny Misael Calvillo-Rodríguez, Cristina Rodríguez-Padilla, Ana Carolina Martínez-Torres

**Affiliations:** Universidad Autónoma de Nuevo León Facultad de Ciencias Biológicas Laboratorio de Inmunología y Virología, San Nicolás de los Garza, Mexico

**Keywords:** cancer, cardiovascular disease, chronic comorbidities, diabetes, immunosurveillance, immunotherapies, NASH, obesity

## Abstract

Immune surveillance is a central function of the immune system that prevents tumor initiation and progression. This process depends on the coordinated activity of innate and adaptive immune responses to recognize and eliminate transformed cells. However, pathological conditions can disrupt immune cell functions, impair immune surveillance, and facilitate tumor immune evasion. Chronic comorbidities, including obesity, type 2 diabetes mellitus (T2DM), non- alcoholic fatty liver disease (NAFLD/NASH), and cardiovascular disease (CVD), are increasingly prevalent among cancer patients and significantly influence tumor-immune interactions. These conditions promote systemic low-grade inflammation, metabolic alterations, immune dysfunction, T cell exhaustion, impaired antigen presentation, and the establishment of tolerogenic tissue microenvironments. Despite their relevance, comorbidities are often underrepresented in preclinical cancer models and insufficiently considered when assessing therapeutic responses. Emerging evidence suggests that chronic comorbidities modulate the efficacy and toxicity of immunotherapies that rely on immune activation. Integrating clinically relevant comorbidities into preclinical cancer models for the development of novel therapeutic strategies will be essential to improve immunosurveillance, limit tumor escape, and optimize personalized cancer immunotherapy outcomes.

## 1 Introduction

Immune surveillance is a fundamental function of the immune system that prevents tumor initiation and progression, thereby maintaining tissue homeostasis through the coordinated activity of innate and adaptive immune responses. However, there has been growing recognition that immunosurveillance represents only one dimension of the complex relationship between the immune system and cancer (1). Tumor cells can progressively evade immune-mediated elimination by reshaping their immunogenic profile and establishing an immunosuppressive tumor microenvironment, a dynamic process known as cancer immunoediting (2). Thus, immune pressure may paradoxically promote the emergence of primary tumors with reduced immunogenicity, allowing them to escape immune recognition and destruction (3). This process reflects the dual role of the immune system in both controlling and shaping tumor evolution and involves sequential phases of tumor elimination, immune-mediated containment of residual malignant cells, and eventual immune escape driven by the selection of less immunogenic tumor variants. Through these mechanisms, cancer immunoediting enables tumor persistence and progression despite ongoing immune pressure.

In patients with chronic degenerative conditions such as obesity, diabetes mellitus, or cardiovascular disorders, persistent low-grade inflammation, oxidative stress, and immune dysregulation may disrupt normal immune homeostasis (4). Obesity, T2DM, non-alcoholic steatohepatitis (NASH), and cardiovascular diseases represent highly prevalent chronic degenerative conditions that share systemic and long-lasting metabolic and inflammatory alterations, which have been consistently associated with increased cancer risk and progression, in contrast to other chronic inflammatory diseases that are often tissue-restricted, episodic, or driven by distinct immunopathological mechanisms (5–8). Also, obesity, T2DM, NASH and cardiovascular diseases are comorbidities that frequently coexist within the same individual and reflect interconnected manifestations of immune-metabolic dysfunction rather than isolated disease entities (5–9). Collectively, these alterations compromise effective cancer immunosurveillance and promote tumor immune escape, facilitating cancer immunoediting and influencing tumor progression and responsiveness to anticancer therapies (10–13).

For these reasons, the objective of this review is to integrate and critically examine key studies investigating cancer within the context of chronic degenerative diseases, including obesity, T2DM, non-alcoholic fatty liver disease (NAFLD/NASH), and cardiovascular disorders, to provide a clearer understanding of how these conditions influence immunosurveillance and cancer immunoediting and the models used for theirs study (**Table 1**).

**Table 1:** Chronic disease models in cancer studies.

Disease	Mouse strain	Chronic disease model	Cancer type	Microenvironment	Therapy tested	References
Obesity	C57BL/6J	HFD	MC38 s.c.	ND	ND	(14)
Obesity	C57BL/6J	HFD	AOM/DSS-induced colorectal cancer	ND	ND	(15)
Obesity	C57BL/6J	HFD	MPA/DMBA-induced breast cancer	ND	ND	(16)
Obesity	C57BL/6J	HFD	LLC s.c.	ND	ND	(17)
Obesity	MMTV-PyMT (Mouse Mammary Tumor Virus–Polyoma Virus Middle T Antigen) transgenic mice	HFD	MMTV-PyMT-induced breast cancer	Immune infiltrate of dysfunctional CD8^+^ T cells and elevated STAT3 activity.	Inhibition of the leptin–STAT3–FAO pathway	(18)
Obesity	KpB	HFD	Ovarian cancer with injection into the ovarian cavity of the AdCre adenoviral vector	ND	ND	(19)
Metabolic syndrome	C57BL/6J	HFD	TRAMP-C1 s.c.	ND	ND	(20)
T2DM	Balb/c	Streptozotocin i.p.	CT26 s.c.	Decrease in the number of CD3+CD4+ and CD3+CD8+ T cells in the tumor microenvironment.	ND	(21)
T2DM	MMTV‐ErbB2/Leprdb/db	NK1Mul/J transgenic strain	Breast cancer in a HER2+ transgenic strain	ND	Metformin/rosiglitazone	(22)
T2DM	MMTV-PyMT	Streptozotocin i.p.	Spontaneous breast cancer due to a transgenic strain	Glycosylated products promote ECM stiffness, which in turn promotes EMT	Insulin	(23)
T2DM	Balb/c	Streptozotocin i.p.	4T1 s.c.	Increased glucose metabolism enzymes (G6PD, GAPDH and ENO1), EMT proteins (vimentin, Snail, β-catenin and TCF8) and proteolytic systems (uPA, uPAR, PAI-1 and MMP-9)	ND	(24)
T2DM	C57BL/6J	HFD + Streptozotocin	KPC or PanO2 orthotopic injection	Senescent endothelial cells in the diabetic tumor microenvironment promote the progression of pancreatic cancer.	Dimagrumab	(25)
T2DM	MKR	Transgenic strain on insulin-resistant FVB/NJ background	Azoxymethane-induced colorectal cancer and dextran sodium sulfate (DSS)	ND	Fecal microbial transplant	(26)
T2DM	KC sobre fondo FVB/N y C57BL/6J	Pancreatic tumor-induced diabetes	Spontaneous pancreatic ductal adenocarcinoma induced with a KC model	Inflammatory; with high macrophage infiltration, fibroblast activation, as well as a loss of β islets and an increase in TGF-β	Neutralizing anti-TGF-β antibody	(27)
T2DM	Balb/c	Streptozotocin i.p.	4T1 Orthotopic Injection	Accumulation of myeloid suppressor cells (MDSCs) and Treg lymphocytes, in addition to IDO- and iNOS-mediated NK cell dysfunction.	ND	(28)
NAFLD	C57BL/6J	Methionine-choline deficient diet (MCD), choline-deficient amino acid-defined diet (CDAA), HFD	Diethylnitrosoamine-induced hepatocellular carcinoma (DENC)	ND	ND	(29)
NASH	C57BL/6J, Balb/c	Choline-deficient L-amino acid-defined diet (CDAA), Western diet.	RIL-175 and CT26 injected into the left hepatic lobe	The frequency of CD8+ T cell tumor infiltration was similar in mice with and without NASH in two tumor models. However, NASH slowed CD8+ T cell motility.	Anti-PD1/ Metformin	(30)
NASH	C57BL/6J	Western diet or HFD deficient in choline	NASH-induced hepatocellular carcinoma	ND	Therapeutic intermittent fasting	(31)
NASH	C57BL/6J	HFD rich in amino acids for choline deficiency	Diethylnitrosoamine-induced hepatocellular carcinoma (DENC)	ND	hNRG4-Fc	(32)
NASH	C57BL/6J	NFD, choline-deficient L-amino acid-defined diet (CDAA), choline-deficient HFD	Diethylnitrosoamine-induced hepatocellular carcinoma (DENC)	ND	ND	(33)
Cardiovascular diseases	C57BL/6J	Transverse aortic constriction (TAC) surgery	MMTV-PyMT-induced breast cancer	ND	ND	(34)
Heart failure	C57BL/6J, Balb/c	Transverse aortic constriction (TAC) surgery	MMTV-PyMT-induced breast cancer	ND	ND	(35)
Heart failure	C57BL/6J	Myocardial infarction (MI) surgery, transverse aortic constriction (TAC), or sham surgery	AOM/DSS-induced colorectal cancer	In the TME, several biological processes related to cell proliferation and cell death were enhanced. This includes the proliferation of mononuclear cells, which was positively regulated.	ND	(36)
Heart failure	C57BL/6J	Myocardial infarction (MI) surgery or sham surgery	LLC s.c.	ND	ND	(37)

## 2 Comorbidities as modulators of immunosurveillance and cancer immunoediting

### 2.1 Obesity

Obesity is defined as a condition characterized by excess adiposity, with or without abnormal distribution or function of adipose tissue and driven by multifactorial causes that remain incompletely understood (38). It is one of the most prevalent non-communicable diseases and represents a major public health concern (39).

**Figure 1:**
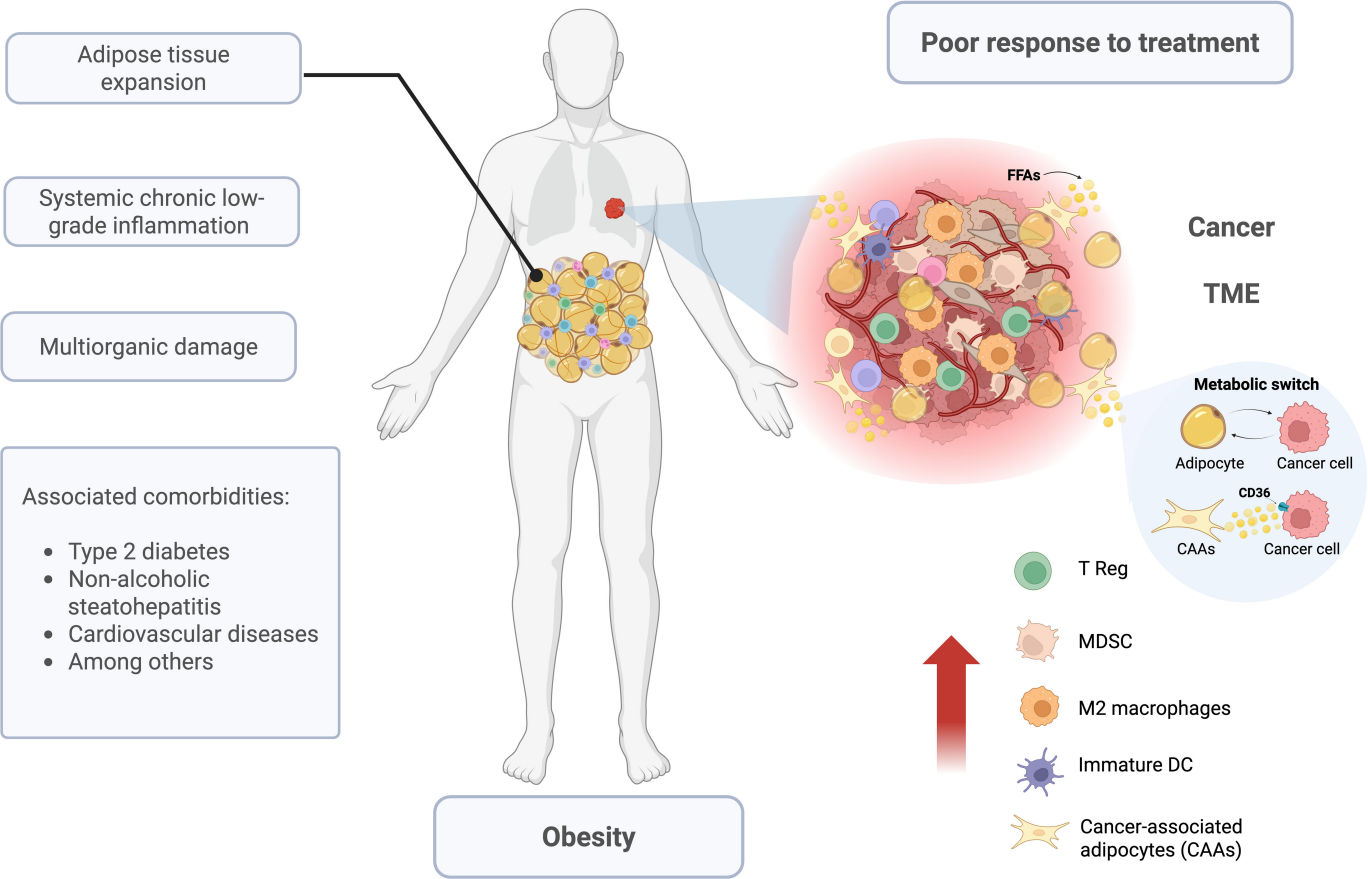
Obesity and cancer. Obesity is defined by adipose tissue expansion, which promotes chronic low-grade inflammation and lipotoxicity with systemic consequences, ultimately leading to multi-organ damage, a condition commonly referred to as clinical obesity. Obesity is strongly associated with multiple comorbidities, including type 2 diabetes mellitus (T2DM), non-alcoholic steatohepatitis (NASH), cardiovascular diseases, and cancer. Within this context, the tumor microenvironment (TME) undergoes profound remodeling, characterized by increased hypoxia, enhanced angiogenesis, and the accumulation of pro-tumoral immune cells and adipocytes. Sustained crosstalk between adipocytes and cancer cells drives metabolic reprogramming of adipocytes toward cancer-associated adipocytes (CAAs). CAAs supply cancer cells with high-energy metabolites and lipid droplets, facilitating a metabolic switch that favors fatty acid β-oxidation, thereby supporting tumor growth, aggressiveness, and reduced therapeutic responsiveness.

Obesity is characterized by a significant inflammatory component that contributes to metabolic and immunologic alterations underlying comorbidities such as insulin resistance, hypertension, atherosclerosis, among others thereby increasing the risk of several types of cancer (5) (**Figure 1**). The relationship between obesity and chronic low-grade inflammation is not entirely understood, but different mechanisms have been proposed. Several studies suggest adipose tissue can secrete more than 50 hormones and signaling molecules referred to as adipokines (40). These adipokines have wide ranging effects in metabolism including satiety, regulation of energy expenditure and energy storage (41). However, in addition to their direct roles in metabolism, adipokines participate in the regulation of immune functions, including cytokine induction, macrophage and neutrophil activation, as well as the recruitment of macrophage and T cells into adipose tissue (42). Among the adipokines produced exclusively by adipose tissue, leptin and adiponectin have been demonstrated to play predominant roles in immune functions (41).

Leptin receptor (Ob-R) is expressed in both innate and adaptive immune cells, including monocytes/macrophages, neutrophils, dendritic cells, NK cells, and T and B cells, and consequently, many immune cells have been shown to be leptin responsive (43). Overall, leptin receptor expression plays a critical role in hematopoietic cell development, immune cell proliferation and survival, and the promotion of pro-inflammatory responses (43, 44). Leptin stimulates cytokine production, such as IL-2, and factors influencing chemotaxis and macrophage activation. However, chronic high levels of leptin, as seen in obesity, affects natural killer (NK) cytotoxicity, neutrophil activation and naïve T cell proliferation (42). Moreover, recent studies suggest that obesity promotes T cell exhaustion through leptin-mediated PD-1 up-regulation (45, 46), underscoring the broad immunomodulatory impact of this adipokine. Given its diverse functions, leptin has been implicated not only in insulin resistance and T2DM but also in cancer (46), reinforcing its predominantly pro-inflammatory role across multiple aspects of immunity and inflammation.

Adiponectin is a high-molecular weight protein that circulates at elevated concentrations and exists in multimeric forms (47). Through its interaction with cell surface receptors, adiponectin promotes fatty acid oxidation (48) and regulates the expression of genes such as CD36 and PPARγ (49). In obesity, adiponectin levels decline, and this reduction contributes to impaired insulin sensitivity, dyslipidemia, altered cholesterol uptake and atherosclerotic plaque formation, endothelial dysfunction, and increased production of adhesion molecules and pro-inflammatory cytokines (50). Dysregulated lipid homeostasis plays a central role in the development of several chronic diseases, which are also characterized by chronic inflammation (51). Adiponectin exerts its effects through AdipoR1 and AdipoR2, which are mainly expressed in monocytes/macrophages, dendritic cells, and T lymphocytes, with lower expression in NK and B cells (52–55). Adiponectin suppresses pro-inflammatory immune responses by inhibiting T cell proliferation and cytokine production, promoting regulatory T cell development, and limiting inflammatory cytokine expression in myeloid cells (56). Additionally, adiponectin inhibits M1-like macrophage activation while favoring M2-like phenotype polarization, thereby exerting potent anti-inflammatory effects (53).

Excessive lipid accumulation, a hallmark of obesity and metabolic disorders, profoundly affects immune cell function and contributes to chronic low-grade inflammation. Lipid overload in macrophages, dendritic cells (DC), and T lymphocytes disrupts their metabolic programming and functional phenotype, leading to impaired antigen presentation, altered cytokine regulation, and a shift toward pro-inflammatory states. In particular, lipid-laden macrophages, often referred to as “foam cells,” exhibit defective phagocytic capacity and sustain tissue inflammation, thereby linking metabolic stress to immune dysregulation. This lipid-induced immune dysfunction plays a pivotal role in establishing the pro-inflammatory and pro-tumorigenic microenvironment characteristic of chronic degenerative diseases and cancer (57). Moreover, the chronic inflammation orchestrated by adiposse tissue macropaghes (ATM) establishes a mutagenic, pro-growth enviorment that favors the acquisition of oncogenic mutations (58). Under conditions such as obesity and chronic low-grade inflammation, DC exhibit altered lipid metabolism, endoplasmic reticulum stress, and reduced expression of costimulatory molecules, which collectively compromise their antigen-presenting capacity (59). As a result, T cell priming becomes inefficient, leading to diminished proliferation and differentiation. This dysfunction not only weakens antitumor immunity but also contributes to immune tolerance and persistent inflammation within metabolic tissues. Such defects in DC–T cell crosstalk exemplify how metabolic stress can reprogram immune responses and promote disease progression (59, 60). Moreover, group 2 innate lymphoid cells (ILC2s) have emerged as a distinct population of innate lymphocytes initially identified in adipose tissue, where they produce high levels of type 2 cytokines, particularly IL-5 and IL-13 (61). Under homeostatic conditions, adipose-resident ILC2s contribute to tissue immune balance by promoting eosinophil recruitment and supporting the polarization of M2-like macrophages, thereby maintaining metabolic and inflammatory homeostasis. Importantly, obesity profoundly alters ILC2 abundance and function, leading to a reduction in their frequency and impaired type 2 cytokine production, which contributes to adipose tissue inflammation, metabolic dysfunction, and immune imbalance (62). Additionally, recent studies have demonstrated that obesity impairs the anti-tumor activity of NK cells, leading to metabolic paralysis and diminished cytotoxic function (63).

Beyond the impairment of NK cell activity, obesity also reduces the abundance of tumor- infiltrating CD8^+^ T cells (64–66). Furthermore, CD8^+^ T cells that do infiltrate tumors in high fat diet (HFD) fed mice were functionally impaired and consequently failed to control tumor growth (67). Consistent with these alterations, obesity is associated with the development of “cold” tumor phenotypes (67), which ultimately compromises the therapeutic efficacy of immunotherapy. Due to T cells are critical for detection and the posterior elimination of malignant cells prior to tumor formation, this lack of vigilant immune surveillance could explain high rates of cancer in people living with obesity (68). However, clinal studies stratifying patients by BMI, have shown that obesity is frequently associated with improved survival following immune checkpoint blockade (ICB) treatment (45, 69). The process by which patients with compromised tumor immunity (e.g. obesity) may paradoxically respond better to immunotherapy is referred to as an obesity paradox and is underlying mechanisms to remain incompletely understood (68).

Recent studies in mouse models of Western diet (WD)–induced obesity have shown that CD8^+^ tumor-infiltrating lymphocytes (TILs) display reduced effector activity and consequently, this alteration was associated with poor tumor control even after ICB immunotherapy (68). These data suggest that TIL dysfunction influences immune surveillance. Furthermore, standard chow and WD–fed mice exhibit distinct immunoediting dynamics that lead to altered tumor phenotypes; consequently, the disruption of immune surveillance contributes to increased tumor outgrowth (68). The same study showed that the protection in lean mice was dependent on a robust antitumor immune response that was reduced in obese mice. In cancer patients living with obesity, the combination of impaired immune surveillance and cancer immunoediting could impact therapeutic outcomes in different ways. Furthermore, WD-fed obese mice exhibit a higher proportion of myeloid-derived suppressor cells (MDSCs) in peripheral blood (70). MDSCs, a type of immature immunosuppressive cells that is produced under abnormal conditions, can suppress anti-tumor immunity through a variety of mechanisms such as prevention of activation signals in CD4+, CD8+ T cells (71), inhibition of T cell activation through production of ROS or PDL-1 (72) or secretion of immunosuppressive factors (TGF-B, IL-10 and IL-12) (73). Those factors allowing tumor cells to escape immune surveillance as well as the promotion of tumor progression (70).

### 2.2 Type 2 diabetes mellitus

Type 2 diabetes mellitus (T2DM) is a chronic metabolic disorder characterized by persistent hyperglycemia, insulin resistance, and a state of low-grade systemic inflammation. Elevated glucose levels, or hyperglycemia, characteristic of T2DM induce an inflammatory response by increasing pro-inflammatory markers such as IL-6, TNF-α, and IL-1β, leading to tissue damage and exacerbating insulin resistance. This process creates a vicious cycle that perpetuates metabolic dysfunction (74, 75). This low and chronic inflammation damages the pancreatic *B-*cells and leads to hyperglycemia. Hyperglycemia causes dysfunction of the immune response, hence patients with T2DM are known to be more susceptible to infections. However, elevated glucose levels can impair functions including chemotaxis and phagocytosis (76). In addition, induced oxidative stress, resulting in overproduction of ROS, exacerbate mitochondrial dysfunction, and in consequence weaken immune system response (77). Chronic systemic inflammation and metabolic dysregulation in T2DM contribute to dysfunction in innate immune cells. Individuals with T2DM experience significant shifts in their immune homeostasis, notably marked by an increase in Th17 cells and higher Th17/Tregs ratio (78). Patients with T2DM display increased circulating levels of C-reactive protein (CRP) and IFN-y evaluated by radio-immunoassay (RIA) (79) and also suppression of Th2 serum cytokines (80). Besides NK cells, which display reduced effector function in individuals with T2DM compared with non-diabetic controls (81).

Hyperglycemia is an important factor for macrophage functional shift towards a pro- inflammatory M1-like phenotype, intensified by an increased production of inflammatory cytokines, which contribute to a persistent inflammatory state (76). T2DM mediated alterations in inflammatory responses may diminish their antitumor immune response, possibly enhancing immunosuppression and reducing immunotherapy effectiveness (82). In patients with T2DM, chronic exposure to high glucose levels alters metabolic landscape of the TME, leading disturbances of immune checkpoints and immune cell exhaustion (83).

Collectively, these metabolic and inflammatory perturbations illustrate how systemic metabolic dysregulation in T2DM reshapes immune cell function and contributes to compromised host defenses.

### 2.3 NAFLD/NASH

Non-alcoholic fatty liver disease (NAFLD) and its progressive form, non-alcoholic steatohepatitis (NASH), are chronic metabolic liver disorders usually linked to obesity, insulin resistance and systemic inflammation. NAFLD is the most common liver disease worldwide (84) and includes a variety of histopathological findings from steatosis with no inflammation to steatosis with multiple levels of inflammation (NASH) (85). The liver is constituted by hepatocytes, the most common type of cell in the liver, resident innate immune cells (e.g. Kupffer cells, NK, among others). After liver damage, other immune cells, including neutrophils and M1-like macrophages are recruited to liver (85). There are multiple “hits” needed for the development and progress of NASH as metabolic factors, immune alterations, including inflammation caused by free fatty acids, cytokines, among others (50). Inflammatory mediators are secreted in response to tissue injury. These mediators activate cellular defense mechanisms and facilitate tissue repair (86). Nevertheless, chronic inflammation can lead to persistent pathological changes, exacerbating tissue damage, and contributing to progression.

The pathogenesis of NASH is associated with a variety of mechanisms including endoplasmic reticulum stress, mitochondrial dysfunction, oxidative stress, and lipotoxicity (87). At the molecular level, hepatocellular lipid accumulation promotes oxidative and endoplasmic reticulum (ER) stress, activating c-Jun N-terminal kinase (JNK) and NF-κB signaling pathways. This triggers the production of pro-inflammatory cytokines such as TNF, IL-1β, and IL-6, leading to recruitment and activation of Kupffer cells, monocyte-derived macrophages, and neutrophils (88, 89). Persistent activation of these myeloid populations drives hepatic inflammation and fibrogenesis through secretion of transforming growth factor-β (TGF-β), which activate hepatic stellate cells (HSCs). This fibrotic remodeling reshapes the hepatic immune microenvironment, promoting an immunosuppressive milieu dominated by Tregs, M2-like macrophages, MDSCs (90).

Kupffer cells, the resident macrophages of the liver, play a central role in the establishment of this tolerogenic state. However, prolonged activation also promotes immune tolerance through expression of PD-L1 and secretion of IL-10 and TGF-β, which suppresses cytotoxic CD8^+^ T cell and NK cell activity (91). The tolerogenic hepatic microenvironment thus becomes a site of impaired antitumor immunity. Both Kupffer cells and Tregs inhibit effective tumor-antigen recognition, while the altered cytokine milieu favors exhaustion of CD8^+^ T cells and metabolic reprogramming toward fatty acid oxidation, limiting effector responses (92). In this context, NAFLD/NASH patients exhibit increased susceptibility to hepatocellular carcinoma (HCC), characterized by immune evasion, chronic inflammation, and altered cross-talk between hepatocytes and immune cells (93). Furthermore, preclinical and clinical data indicate that NASH- associated hepatocellular carcinoma may respond less effectively to immune checkpoint inhibitor therapy (92).

### 2.4 Cardiovascular diseases (atherosclerosis, heart failure, etc.)

Systemic and local inflammation have a central rol in the development and progression of cardiovascular disease (CVD) (94). Over the last decades, studies have shown that atherosclerosis is a low-grade inflammatory disease (95).

Several acute or chronic conditions can activate endothelial damage and dysfunction; this promotes a vascular low-grade inflammatory response and consequently progression of atherosclerosis (96). All phases of atherosclerosis, from retention of lipoproteins within the arterial wall, to plaque development and subsequent rupture, involve a complex network. This complex network includes innate and adaptative immune systems (97). In healthy conditions the endothelium has anti-inflammatory properties, CV risk factors, bacterial or viral infection and environmental stress, resulting in loss of anti- inflammatory properties, damage of endothelial junctions and an increase in the permeability to macromolecules, therefore changes lead to a subendothelial accumulation of cholesterol- containing lipoproteins which triggers low-grade inflammatory response (98).

Atherosclerosis is fundamentally driven by chronic inflammation and extensive reprogramming of the monocyte–macrophage compartment. Circulating monocytes infiltrate the arterial intima, where they encounter oxidized LDL and cholesterol crystals that activate pattern-recognition receptors such as TLR2, TLR4, and the NLRP3 inflammasome (99, 100). This activation triggers NF-κB– and caspase-1–dependent signaling, leading to secretion of IL-1β and IL-18 and sustaining a pro-inflammatory milieu. Under these conditions, macrophages adopt an M1-like glycolytic phenotype characterized by the accumulation of metabolites such as succinate and citrate that reinforce inflammatory gene expression (101, 102). In contrast, inflammation resolution requires M2-like macrophages that rely on oxidative phosphorylation and fatty acid oxidation; however, mitochondrial dysfunction and lipid overload in advanced plaques disrupt this balance, leading to metabolic inflexibility and persistent inflammation (57).

Myocardial injury triggers a robust inflammatory response driven by the release of damage- associated molecular patterns (DAMPs) from necrotic cardiomyocytes. These signals activate pattern-recognition receptors, including Toll-like receptors, leading to NF-κB–dependent proinflammatory pathways and the production of cytokines and chemokines that recruit immune cells to the injured tissue. While this response is essential for clearing dead cells and initiating repair, persistence or dysregulation promotes adverse remodeling and contributes to the progression of heart failure. These mechanisms have been well characterized in the context of myocardial injury, repair, and remodeling (103).

Cardiovascular disease and cancer share several pathways, including PI3K–AKT–mTOR, NF-κB, and JAK–STAT3 (104). Persistent systemic inflammation and oxidative stress further reshape the tumor microenvironment by impairing vascular perfusion and promoting hypoxia, which limits immune-cell infiltration and reduces responses to immunotherapy (105). Thus, chronic cardiovascular inflammation extends beyond vascular pathology to establish a systemic inflammatory milieu that weakens tumor immunosurveillance.

## 3 Common mechanisms of altered immunosurveillance and cancer immunoediting

These comorbidities (obesity, T2DM, NASH, and cardiovascular diseases) share several common risk factors including age, diet, smoking, pollution and other lifestyle or environmental exposures that are also linked to cancer risks, which make these interactions quite complex as on the one hand they can independently contribute to one or another in an independent way but also these can also influence carcinogenesis.

**Figure 2:**
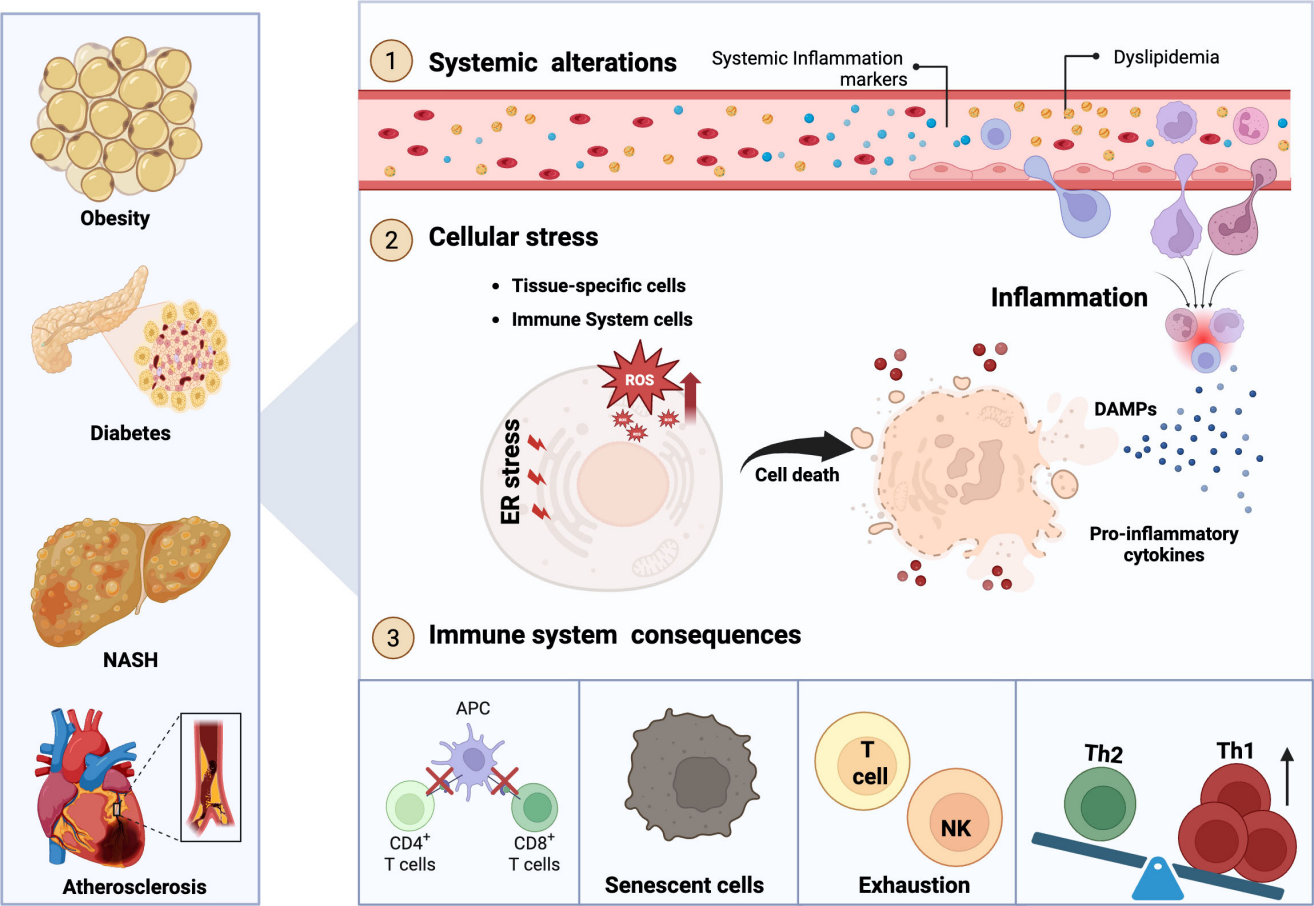
Obesity, T2DM, NASH, and atherosclerosis share common systemic, cellular, and immune system alterations. Metabolic and inflammatory diseases such as obesity, T2DM, NASH, and atherosclerosis share common systemic and cellular disturbances that converge on immune dysfunction. Systemically, these conditions are characterized by dyslipidemia and persistent release of pro-inflammatory cytokines, sustaining chronic low-grade inflammation. At the cellular level, increased oxidative stress, reactive oxygen species (ROS) accumulation, and endoplasmic reticulum stress promote cell death. The consequent release of damage-associated molecular patterns (DAMPs) and inflammatory mediators drives continuous immune cell recruitment, perpetuating unresolved inflammation. Over time, this inflammatory milieu leads to immune impairment, including defective antigen presentation, immune cell senescence, exhaustion of T and NK cells, and disrupted immune homeostasis with a bias toward Th1-type immune responses.

Also, obesity, T2DM, NASH, and cardiovascular diseases are tightly interconnected as they share multiple common features at both systemic (through metabolic and inflammatory pathways) and cellular levels, ultimately exerting a significant impact on the performance of the immune system. Among the shared systemic effects is the presence of inflammatory markers (e.g., cytokines, C-reactive protein, among others) (39, 106–108), as well as dyslipidemia (109). These systemic alterations lead to cellular stress affecting both tissue-specific cells involved in the pathology and tissue-resident immune cells. This cellular stress is characterized by endoplasmic reticulum stress and increased production of reactive oxygen species (ROS) (110), culminating in cell death of both tissue-specific and immune cells. Such cell death is associated with the release of DAMPs, the presence of pro-inflammatory cytokines, and activation of inflammatory processes (111–113), thereby promoting the recruitment of peripheral immune cells (e.g., neutrophils, monocytes, and lymphocytes). This persistent inflammatory state results in immune system dysfunction, including reduced antigen-presenting capacity, accumulation of senescent cells, exhaustion of T and NK lymphocytes, and a shift in the Th1/Th2 ratio toward Th1 dominance. Ultimately, these alterations impair immunosurveillance, facilitating immune evasion, and immunoediting cancer cells (**Figure 2**). Impaired immunity is associated with chronic-degenerative diseases that extend to T cell dysfunction in the tumor microenvironment (68, 114, 115).

All these alterations in immune surveillance could influence the selective pressure on tumor cells. In individuals’ effective immune surveillance, selective pressure applied by the immune system can promote the development of immune evasion mechanisms in tumor cells (116). This leads to the formation of progressor mutations that confer a selective advantage for cancer cells. When immune surveillance is compromised, the selective pressure that normally shapes tumor evolution is diminished, allowing the persistence and expansion of malignant clones that would otherwise be eliminated in immunocompetent hosts.

This decreased pressure may allow the survival of more immunogenic tumor cell populations within the context of cancer immunoediting. However, increased tumor immunogenicity does not necessarily translate into effective immune-mediated tumor control. Antitumor immunity is severely limited by persistent immunological dysfunction, altered cytokine networks, and compromised effector cell function in chronic inflammatory and metabolically dysregulated conditions. As a result, these patients have a higher incidence of cancer and frequently poorer clinical outcomes despite the potential presence of immunogenic malignant cells. This indicates that host immunological competence is more important than tumor immunogenicity alone.

These altered immune landscapes can compromise the efficacy of anticancer immunotherapies that rely on functional immune activation, while simultaneously increasing the risk of treatment-related toxicity and resistance. These observations underscore the need to reinterpret classical immunoediting models within the context of chronic disease associated immune dysfunction and provide a rationale for investigating how metabolic and inflammatory comorbidities reshape tumor–immune interactions and therapeutic responsiveness. To date, and to the best of our knowledge, it remains unexplored how impaired immune surveillance reshapes tumor cell immunogenicity and which compensatory mechanisms are engaged as a consequence.

## 4 Implications for cancer immunotherapy

Several studies have evaluated the efficacy and safety of immunotherapies in patients with cancer and other chronic underlying diseases, demonstrating positive effects in a limited number of cases, along with a substantial number of adverse effects and an overall limited therapeutic response (**Table 2**).

**Table 2:** Association between chronic comorbidities and cancer in clinical trials.

Disease	Determination	Type of participants	Cancer type	Correlation	Therapy	References
Obesity	BMI and anthropometric measures	n=67 142 postmenopausal women ages 50 to 79 years	Breast cancer	Obesity is associated with increased invasive breast cancer risk in postmenopausal women.	Postmenopausal hormone therapy.	(117)
Obesity	BMI	n=404,576 men and n=495,477 women who were free of cancer at enrollment	Different types of cancer	Increased body weight was associated with increased death rates for all cancers combined.	ND	(118)
Obesity	BMI	n= 18,880 men age 55 years and older with a normal digital rectal exam (DRE) and prostate specific antigen (PSA), and no history of prostate cancer.	Prostate cancer	Circulating serum biomarkers may modify the association of obesity with prostate cancer risk.	ND	(119)
Obesity	BMI	n= 6,729 men who underwent at least one on-study biopsy for prostate cancer.	Prostate cancer	Obesity is associated with decreased risk of low-grade and increased risk of high-grade PC.	ND	(120)
Obesity and metabolic syndrome	blood glucose, triglycerides, HDL-cholesterol, blood pressure, waist circumference, HOMA-IR and BMI	n = 68,132 women between the ages of 50 and 79.	Breast cancer	Both obesity and metabolic dysregulation are associated with breast cancer risk	ND	(121)
Obesity and metabolic syndrome	BMI, waist circumference, blood pressure, cholesterol.	n= 68,132 postmenopausal women, without prior breast cancer and with normal mammogram.	Breast cancer	MetS and obesity status have independent, but differential, adverse associations with breast cancer receptor subtypes and breast cancer mortality risk.	ND	(122)
T2DM and obesity	BMI and anthropometric measures	n=10,258 participants who all had cancer presence or absence determined by prostate biopsy.	Prostate cancer	Obesity increases the risk of high-grade prostate cancer, and this relationship is independent of the lower risk for prostate cancer among men with T2DM.	Finasteride	(123)
T2DM	ND	Medication use data from n= 122 patients with non-muscle-invasive bladder cancer treated with BCG.	Non-muscle-invasive bladder cancer	Metformin use was associated with improved oncological outcomes in patients with non-muscle-invasive bladder cancer treated with intravesical BCG.	Metformin, BCG	(124)
T2DM	BMI and circulating levels of glycated hemoglobine (HbA1c)	n=44,352 men with prostate cancer and n=221,495 age-matched men from the general population.	Prostate cancer	There was a reduced risk of being diagnosed with prostate cancer among men with T2DM.	Glucose-lowering therapy	(125)
Diabetes mellitus	By past medical history or insulin and oral hypoglycemic medication.	3,759 patients with high-risk stage II and stage III colon cancer.	Colon cancer	Patients with T2DM and high-risk stage II and stage III colon cancer experienced a significantly higher rate of overall mortality and cancer recurrence.	Insulin and oral hypoglycemic medication	(126)
T2DM	BMI	n= 55,215 men aged 50–69 years with raised PSA levels, diagnosed with prostate cancer or diabetes history.	Prostate cancer	T2DM is associated with a decreased risk of PSA-detected prostate cancer.	ND	(127)
T2DM	Past medical history	Evaluation of self-reported diabetes history (n=62) among 504 participants that developed exocrine pancreatic cancer.	Pancreatic cancer	T2DM is associated with worse survival among patients with pancreatic cancer.	ND	(128)
T2DM	ND	n=493 704 men and n=502 139 women without colon cancer were followed from 2003 to 2005.	Colon cancer	T2DM remains a significant risk factor for colon cancer. Metformin may protect against colon cancer.	Metformin	(129)
T2DM	ND	n= 80 female patients with breast cancer with T2DM were randomly assigned to receive chemotherapy only or chemotherapy plus dexrazoxane (DEX).	Breast cancer	DEX protects against cardiotoxicity induced by chemotherapy in patients with breast cancer with concurrent T2DM.	Epirubicin, cyclophosphamide, DEX	(130)
NAFLD	Hypercholesterolemia	One hundred five consecutive patients with HCC were studied; mean age was 59 years.	Hepatocellular carcinoma	Surveillance of patients with cirrhosis can detect HCC at an earlier stage, possibly leading to an improved survival.	Possible liver transplantation or surgical resection	(131)
NASH/NAFLD	Aspartate aminotransferase, alanine aminotransferase	Patients treated with atezolizumab plus bevacizumab or lenvatinib or sorafenib as first line treatment for advanced or intermediate HCC.	Hepatocellular carcinoma	Treatment with lenvatinib is associated with a significant survival benefit compared to atezolizumab plus bevacizumab, in particular in patients with NAFLD/NASH-related HCC.	Lenvatinib, atezolizumab plus bevacizumab	(132)
Atherosclerosis	ND	n=10 061 patients with atherosclerosis who had had a myocardial infarction and were free of previously diagnosed cancer.	Lung cancer	Therapy with canakinumab could significantly reduce incident lung cancer and lung cancer mortality.	Canakinumab	(133)

### 4.1 Immune checkpoints inhibitors

The use of immune checkpoint inhibitors (ICIs) helps overcome immune tolerance and promotes antitumor responses but may also induce a series of immune-related adverse events (irAEs). Regarding their use in patients with underlying chronic inflammation, such as those with obesity, recent studies have surprisingly shown that individuals living with obesity exhibit higher response rates to ICIs as anti PD-1/PDL-1 in non-small cell lung cancer (NSCLC) (134), even though obesity has been associated with poorer prognosis in cancer patients (135). Tumors developed in obese patients were shown to have different TME, with more exhausted T cells and higher expression of PD-1 (136), features that can be leveraged by anti PD-1/PDL-1 to improve antitumor efficacy. Unfortunately, this effect has only been observed in pre-clinical obesity models, in which no additional comorbidities are present. Paradoxically, recent studies have shown that patients with obesity exhibit improved overall survival (OS) and progression-free survival (PFS) following ICB treatment compared with patients who are overweight or of normal weight in certain cancer types, including NSCLC and renal cancer (137). Similarly, other studies have reported that patients with obesity treated with PD-1/PD-L1 inhibitors have a higher objective response rate (ORR) than non-overweight patients. However, these patients also experience higher rates of irAEs (138). Notably, this positive effect is not consistently observed among all immune checkpoint–based therapies.

Regarding the use of ICIs in patients with other chronic degenerative diseases, the response has not been that favorable. In patients with T2DM, ICIs have shown reduced efficacy in lung cancer, suggesting that T2DM may impair the patient’s ability to mount an effective antitumor immune response (139), due to defects in immune cell migration to sites of inflammation, decreased ROS production, or impaired phagocytosis (140). Moreover, ICI therapy has beenreported to cause damage to pancreatic islet cells, leading to ICI-induced type 1 DM (141). Additionally, patients taking glucose-lowering medications such as metformin have been shown to experience increased risk of death and disease progression with the use of ICIs (83).

Patients with NAFLD have also been found to respond less effectively to ICI treatment (142). Furthermore, meta-analyses have shown increases in liver damage enzymes ALT and/or AST in 2–5% of patients receiving ICI therapies (143). In addition to the damage caused by ICIs, similarities exist between the pathological mechanisms underlying NASH development and those responsible for ICI-induced hepatotoxicity (142).

Experimental studies have demonstrated an increase in exhausted CD8+ PD-1+ T cells in NASH models, with NASH severity correlating with PD-L1 expression in hepatocytes and non-parenchymal cells (92). Recent studies have shown that in murine models using C57BL/6 mice fed a choline-deficient high-fat diet (CD-HFD) for 13 months to induce liver tumors with genetic alterations resembling human NAFL-HCC or NASH-HCC, animals exhibit a poor response to anti–PD-1 therapy despite an increase in CD8+ T cells. Notably, these mice also display increased fibrosis and liver injury, along with a higher incidence of hepatocellular carcinoma, without a reduction in tumor size (92). In contrast, in a murine model of liver cancer without NASH, C3H/HeJ mice bearing diethylnitrosamine-induced (DEN-induced) tumors respond favorably to anti–PD-1 therapy, showing tumor regression (144). Consistently, phase III clinical data indicate that patients with NASH-derived HCC treated with anti–PD-1 or anti–PD-L1 exhibit reduced overall survival (92), suggesting that NASH-HCC may be associated with reduced responsiveness to immunotherapy. A potential benefit of ICI use is its ability to reduce liver damage when administered in combination with anti-TNF or anti-CD8 antibodies in patients with NASH, thereby decreasing the incidence of HCC (142).

In the cardiovascular system, although ICIs activate antitumor immunity, they disrupt immune homeostasis (145). Recent studies have shown that patients receiving ICIs have an increased risk of developing atherosclerosis (146). Additionally, ICI therapy has been associated with significant progression in the non-calcified volume of atherosclerotic plaque and with greater overall plaque progression (147), contributing substantially to reduced survival and increased 30-day mortality due to arterial events (146).

### 4.2 Cytokines

The use of cytokines in patients with cancer and underlying conditions that exacerbate the inflammatory state has not been yet studied. However, studies have shown that the use of cytokines in different comorbidities may not be entirely beneficial. Therapies such as IL-2 combined with high-dose IFN have been tested; however, they result in cytokine storm and multiorgan failure in patients living with obesity (148), thereby limiting their clinical use. Monoclonal agonistic antibodies targeting CD40 have also been evaluated in combination with IL-2. This therapy induces a strong antitumor response and is well tolerated in young murine models (149). However, in diet-induced obese mouse models, its administration triggers a cytokine storm, organ damage, and increased lethality (150). In the context of atherosclerosis, high doses of IFN-α have been shown to increase plasma levels of cholesterol and triglycerides, promote lipid uptake by macrophages, enhancing foam cell formation, and inhibit the activation and proliferation of Treg lymphocytes (151). Unfortunately, there are still no studies evaluating cytokine-based therapy in patients with cancer with other comorbidities; therefore, there is an urgent need for further research to assess the safety and efficacy of these therapies.

### 4.3 Adoptive cell therapy

There is increasing evidence supporting the therapeutic potential of dendritic cell (DC)-based vaccines in cancer treatment (152). It is reported that monocyte-derived DCs (Mo-DCs) obtained from patients with T2DM and obesity exhibit significant phenotypic and functional impairments when compared to Mo-DCs from individuals with obesity alone or healthy controls (153). These altered Mo-DCs expressed higher levels of integrins such as CD18 and CD11c, which can promote leukocyte adhesion to the vascular endothelium and exacerbate systemic inflammation. In addition, hyperglycemia enhances the expression of adhesion molecules by modulating immune cell recruitment and contributing to immune dysregulation.

Natural killer (NK) cells play a central role in tumor immunosurveillance and are increasingly studied in adoptive cancer immunotherapy. However, there is only evidence of the impact of T2DM on these vaccines at the preclinical level. According to recent studies, adoptive NK cell transfers derived from streptozotocin (STZ)-induced diabetic Balb/c mice were significantly less effective at controlling Panc1 tumor growth compared with NK cells obtained from normoglycemic mice (154).

Chimeric antigen receptor (CAR) T cell therapy has emerged as a new therapeutic pillar for hematologic malignancies and is currently under investigation in solid tumors. Under certain clinical conditions (such as high tumor burden, concomitant or prior infections, and acute inflammation) this therapy can be associated with distinct toxicities, most notably cytokine release syndrome (CRS) and immune effector cell–associated neurotoxicity syndrome (ICANS) (155). ICANS typically occurs following CRS, which is considered a critical initiating event (156).

Recent findings indicate that body composition, particularly increased visceral adipose tissue (VAT), correlates with greater CRS severity and earlier onset; however, these parameters do not appear to influence ICANS incidence or severity (155). Consistently, hyperlipidemia significantly increases the risk of severe CRS and that overweight patients exhibit reduced circulating CAR T cell levels after infusion (157). Although CRS can result in cardiac dysfunction and hemodynamic collapse (negatively affecting clinical outcomes and survival) baseline echocardiographic parameters (including left ventricular ejection fraction and global longitudinal strain) do not predict the development of clinically significant CRS in patients receiving CD19 CAR T cell therapy (158). Cardiac toxicity is uncommon, with an incidence of approximately 4.5%, and occurs in patients experiencing moderate to severe CRS, suggesting that cardiac dysfunction is driven by systemic inflammation rather than pre-existing cardiac impairment.

### 4.4 Monoclonal antibodies

Adipose tissue expansion in obesity leads to increased hypoxia, driving the upregulation of pro-angiogenic factors such as VEGF and revealing a potential target for monoclonal antibody-based therapies (159). However, anti-VEGF treatment shows reduced efficacy in murine models of diet-induced obesity (DIO) (160). This reduced response has been linked to IL-6 overproduction in the tumor microenvironment of patients with obesity and breast cancer, which further induces VEGF expression, promoting tumor growth and resistance to anti-VEGF therapy (161). Supporting this mechanism, combined inhibition of IL-6 and VEGF significantly reduces tumor growth and metastasis in DIO mice (160). Notably, IL-6 blockade does not enhance therapeutic responses in lean animals, underscoring IL-6 as a key mediator of obesity- associated resistance to anti-VEGF treatment.

On the other hand, a major drawback of anti-VEGF antibody therapy is its tendency to induce atherosclerosis (151). Preclinical studies have shown that administration of anti-VEGF antibodies impairs endothelial function and increases atherosclerotic lesion development by up to 33% in Apoe-/- mice (162). Although direct experimental evidence is still limited, a shift toward a pro-inflammatory Th1- type response and a reduction in Tregs has been proposed to contribute to the pro-atherogenic properties of VEGF-blocking antibodies (163). Additionally, HER2-targeted monoclonal antibodies such as trastuzumab carry the risk of inducing heart failure in up to 20% of treated patients (164), with a nearly four-fold higher incidence than that associated with conventional chemotherapy (165). Unfortunately, no studies have yet evaluated the clinical benefits of these therapies in patients with underlying comorbidities such as T2DM, cardiovascular disease, or NASH.

## 5 Conclusion and future directions

**Figure 3:**
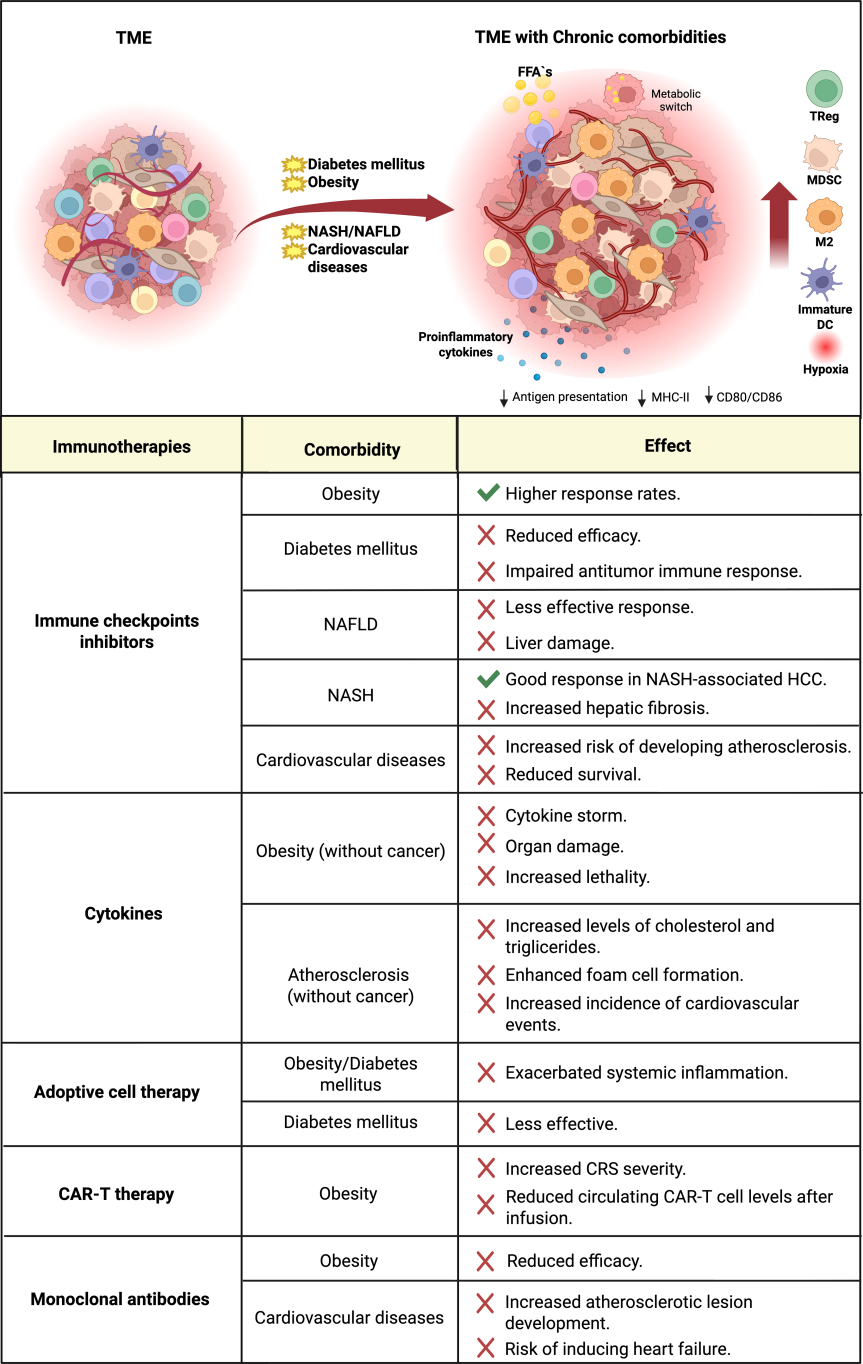
The tumor microenvironment in the presence of chronic comorbidities and its implications for the efficacy of cancer immunotherapies. The tumor microenvironment in the context of chronic diseases such as obesity, T2DM, NASH/NAFLD, and cardiovascular diseases is characterized by increased infiltration of immunosuppressive cells, hypoxia, release of pro-inflammatory cytokines, and lipotoxicity affecting immune system cells. Lipid overload in antigen-presenting cells results in reduced expression of costimulatory molecules and impaired phagocytic capacity, leading to defective antigen presentation. In T lymphocytes, it induces cellular exhaustion accompanied by increased PD-1 expression. This immune dysfunction is key to the establishment of a pro-inflammatory and pro-tumorigenic tumor microenvironment, as well as to reduced immune surveillance. The efficacy of immunotherapies is profoundly influenced by the remodeling of the tumor microenvironment and the immune dysfunction observed in patients with chronic comorbidities. These alterations can compromise therapeutic responses by limiting effective immune cell activation, infiltration, and persistence within the tumor. In some cases, however, the exacerbated inflammatory state may enhance responsiveness to certain immunotherapeutic approaches. Nevertheless, this imbalance is often accompanied by an increased incidence of immune-related adverse events, reflecting an unstable immunological balance. TME, tumor microenvironment; NASH, non-alcoholic steatohepatitis; NAFLD, non-alcoholic fatty liver disease; FFA´s, free fatty acids; TReg, regulatory T cells; MDSC, myeloid-derived suppressor cells; DC, dendritic cell; HCC, hepatocellular carcinoma; CRS, cytokine release syndrome.

Chronic conditions such as obesity, T2DM, NASH, and cardiovascular diseases continue to rise worldwide. Their increasing prevalence among cancer patients has important implications, as these comorbidities can substantially influence the immune system, impacting cancer immunosurveillance, cancer immunoediting, and modifying treatment outcomes (**Figure 3**). This underscores the need to assess and investigate how chronic comorbidities affect cancer immunosurveillance, the cancer immunoediting, and the efficacy of immunotherapies, given their impact on tumor progression, treatment response, quality of life, and overall mortality risk.

Although several studies have examined one or more of these chronic conditions in the context of cancer, including their effects on immunosurveillance and immunotherapy outcomes, most research still fails to adequately consider these comorbidities when evaluating such parameters. Consequently, there remains a pressing need for integrative immuno-oncology approaches that explicitly incorporate chronic comorbidities into experimental design and interpretation. Chronic conditions can reshape immune landscapes, leading to tumor behaviors that differ markedly from those observed in tumor models lacking these conditions. This is particularly relevant given that most studies on the tumor microenvironment, tumor progression, and responses to immunotherapy are conducted in young, otherwise healthy animal models. Addressing comorbidity-related immune alterations is therefore essential not only for improving therapeutic outcomes but also for advancing our understanding of these diseases and possible unexpected negative outcomes.

Future strategies should combine cancer-targeted approaches with experimental models that integrate relevant comorbidities. Also, additional therapeutic opportunities can be taken into consideration; these can include metabolic, anti-inflammatory, and lifestyle interventions. Also, microbiota modulation could result in interesting outcomes. Such efforts may facilitate the development of host-directed interventions capable of enhancing cancer cell immunogenicity, restoring effective immunosurveillance, and promoting robust antitumor immune responses.
